# Cobalt-conjugated carbon quantum dots for in vivo monitoring of the pyruvate dehydrogenase kinase inhibitor drug dichloroacetic acid

**DOI:** 10.1038/s41598-022-22039-w

**Published:** 2022-11-12

**Authors:** Jiko Raut, Md Majharul Islam, Rinchen D. Sherpa, Biraj Sarkar, Shanti M. Mandal, Subhra P. Hui, Sukhendu Mandal, Prithidipa Sahoo

**Affiliations:** 1 Department of Chemistry, Visva-Bharati University, Santiniketan, 731235 India; 2 Department of Microbiology, University of Calcutta, Kolkata, 700019 India; 3 S. N. Pradhan Centre for Neurosciences, University of Calcutta, Kolkata, 700019 India; 4 Central Research Facility, Indian Institute of Technology Kharagpur, Kharagpur, 721302 India

**Keywords:** Chemistry, Materials science, Nanoscience and technology

## Abstract

Dichloroacetic acid (DCA), an organohalide that present in environmental sample and biological systems, got high attention for its therapeutic potential as the inhibitor of pyruvate dehydrogenase kinase (PDK), elevated in obesity, diabetes, heart disease and cancer. Herein, we developed a Cobalt conjugated carbon quantum dots (N-CQDs/Co) that selectively detect DCA by fluorescence “turn-on” mechanism. Utilizing TEM, DLS, UV–vis and fluorescence spectroscopy, the mechanism has been thoroughly elucidated and is attributed to disaggregation induced enhancement (DIE). The limit of detection of the N-CQDs/Co complex is 8.7 µM. The structural characteristics and size of the N-CQDs and N-CQDS/Co complex have been verified using FT-IR, XPS, HRTEM, DLS, EDX have been performed. Additionally, the complex is used to specifically find DCA in the human cell line and in zebrafish.Journal instruction requires a city for affiliations; however, these are missing in affiliation [4]. Please verify if the provided city is correct and amend if necessary.Kharagpur is the city. The address is okay.

## Introduction

Dichloroacetic acid (DCA) is an organohalide that can be produced from the chlorinated drinking water, disinfectants, metabolites, or by-products of various industrially used solvents and chlorinated drugs^1, 2^. Despite its accumulation in groundwater and potential health hazard, no assessment of DCA exposure levels was investigated worldwide in connection with human or environmental health^3^. Absorption of DCA into the body may occur through the skin when bathing or swimming in chlorinated pools, or through the air^4^. With an alarming concern regarding human exposure to DCA from environmental sources, it is also noteworthy that DCA has been used as a drug component for about half a century to treat a variety of metabolic, cardiovascular and cerebrovascular disorders, hypercholesterolemia, lactic acidosis, and cancer^1^. In the late 1950s, diisopropylammonium dichloroacetate (DIPA) was used to treat diabetes mellitus, liver dysfunction, or vascular diseases, and later in 1970 DCA was found as the metabolically active moiety of DIPA which has been used as the sodium salt in experimental and clinical studies since then^5^. Sodium DCA has been given intravenously and orally in doses ranging from 25 to 100 mg/kg for periods extending from a few days to more than 10 years^6, 7^. Prolong exposure of DCA leads to the reversible peripheral neuropathy observed in rodents, dogs and humans. In humans, DCA at clinically relevant levels (mg/kg/day) reduces the expression and activity of glutathione transferase zeta (GSTz1-1), altering tyrosine metabolism and leading to hepatic and neurological damage.

Despite of its possible toxicity, the medicinal potential of DCA is still being researched^8–12^. DCA, a pyruvate dehydrogenase kinase inhibitor (PDK), increases pyruvate dehydrogenase (PDH) activity by enhancing mitochondrial pyruvate absorption and promoting glucose oxidation during glycolysis. DCA blocks PDK that enhances the rate of pyruvate conversion which in turn increase production of adenosine triphosphate (ATP), and thereby prevents ischemia-induced neuronal death^13^.

The pharmacological profile of DCA has sparked a fascinating debate between environmental toxicologists who believe it is a human threat, on the contrary, clinical scientists who are looking into its therapeutic possibilities. Moreover, there is a rising argument about the relevance of DCA to human cancer, where it is seen as either a likely cause or a potential cure^14^. DCA is still an investigational medicine that has yet to be approved by the FDA to treat any condition^15^. Thus, the tracing of DCA in biological or environmental samples is very much important.

**Figure 1:**
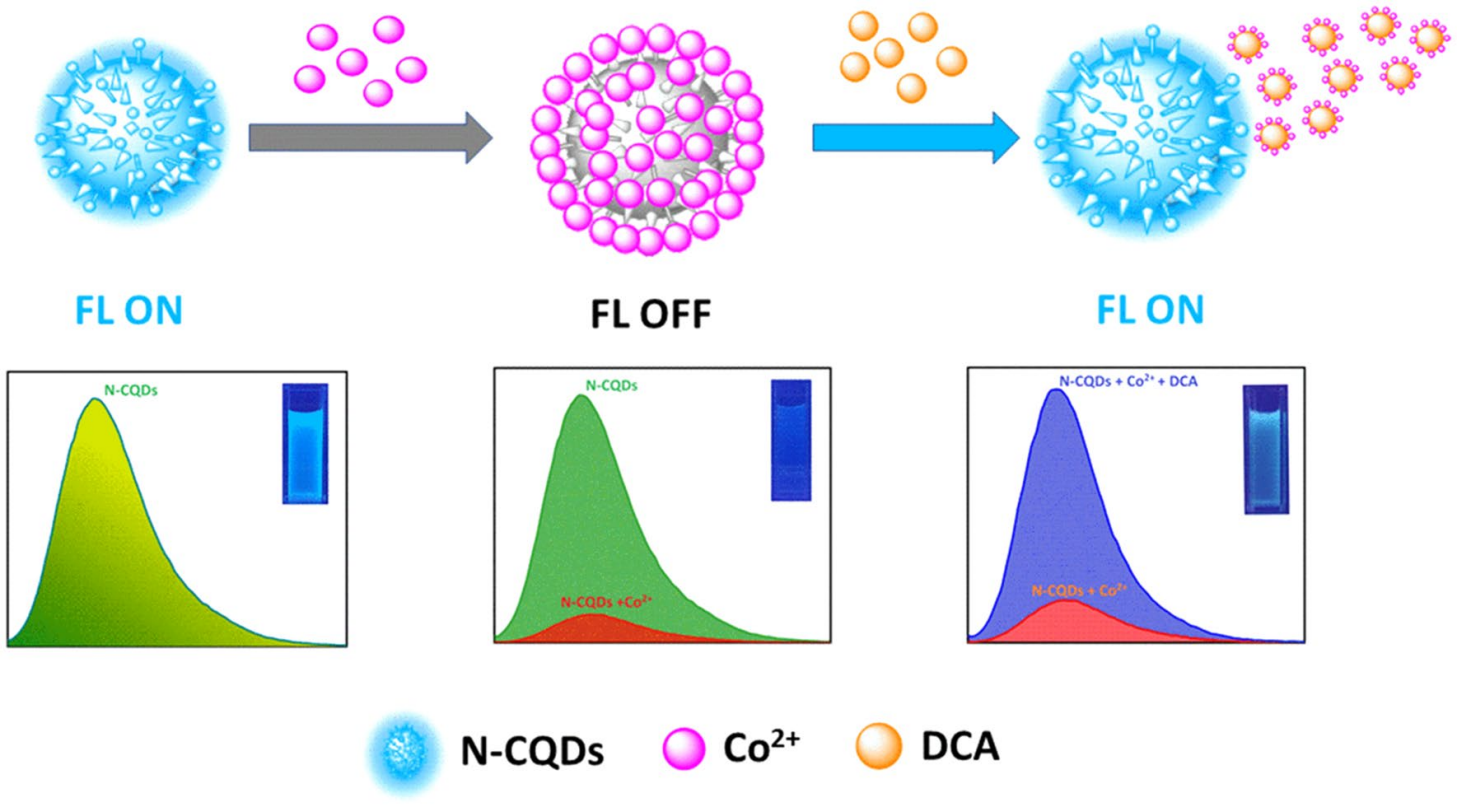
Schematic illustration of a switchable fluorescent N-CQDs for the detection of DCA based on aggregation and disaggregation mechanism.

There is no detection or estimation strategy of DCA available to date that can help to monitor levels of DCA in tissues or plasma. Presently, quantum dots (QDs) have been widely used as fluorescent nanoprobes for bioimaging and sensors due to their outstanding photostability, low toxicity, favorable biocompatibility and strong water solubility. Because of the spatial confinement of QDs nanostructures, radiative recombination rates are increased while nonradiative recombination rates are decreased, resulting in a significant increase in fluorescence efficiency. As a result, developing quantum dots as novel fluorescence platforms can enable advanced applications in biological labelling, diagnostics, medicine and environmental monitoring devices. Our goal in this study is to identify DCA in biological systems that will aid future research on the details pharmacokinetics of DCA. We developed a water-soluble N-CQDs/Co complex that can selectively recognize DCA in in vitro and in vivo systems through turn-on fluorescence signaling. Cobalt (2+) is utilized to quench the fluorescence of N-CQDs while DCA restores the quenched fluorescence of the N-CQDs, resulting in a favorable “turn on” state (Fig. 1). Our proposed fluorescent “off–on” sensor for DCA detection is simple and quick and could be useful for monitoring DCA in clinical research. The cell permeability and specificity of detection of the N-CQDs/Co make it a reliable sensor for detecting DCA in in-vitro and in-vivo cell lines or zebrafish model.

Figures are not in sequential order, hence it has been renumbered in artwork and citations. Kindly check and confirm.Thank you very much for the correstions. Now it is absolutely OKAY.Please confirm the section headings are correctly identified.Yes, it is okay

## Results and discussions

### Characterization

**Figure 2:**
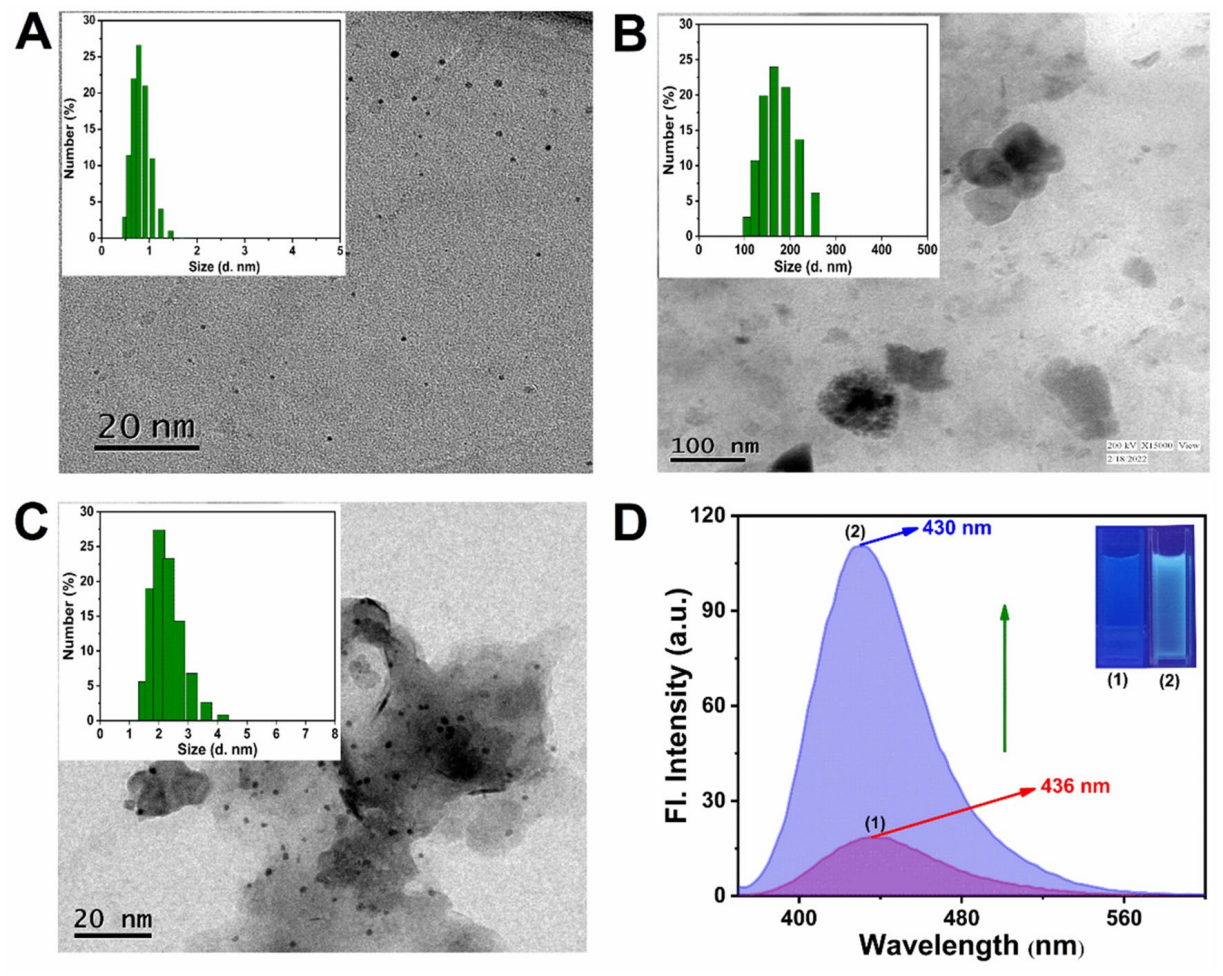
TEM images of (**A**) N-CQDs (**B**) Aggregated N-CQDs by Co^2+^ (**C**) Disaggregated N-CQDs by DCA. Inset: the corresponding hydrodynamic diameter measured by DLS (**D**) Fluorescence emission spectra of N-CQDs/Co complex in the (1) absence and (2) presence of DCA. Inset: corresponding fluorescence images under UV light (365 nm).

Transmission electron microscopy (TEM, HRTEM) and dynamic light scattering (DLS) were used to examine the morphology of the fabricated N-CQDs. The TEM image (Fig. 2A) supports the creation of spherical and monodispersed N-CQDs with particle size range from 1 to 2 nm, which corroborated with the DLS result (Inset in Fig. 2A). In HRTEM image (Fig. S1), the presence of significant lattice fringe suggests that the N-CQDs are crystalline. The FT-IR spectrum of synthesized N-CQDs is shown in Fig. S2. The stretching and bending vibrations coming from FT-IR spectroscopy clearly explain the presence of –OH/–NH, C–H, C=N, C–O, N–H and C=O amide group. It also confirms the nitrogen doping on N-CQDs^16–19^. X-ray photoelectron spectroscopy (XPS) was carried out to gain additional structural understanding. The full XPS spectrum of N-CQDs & N-CQDs/Co complex (Fig. S3) shows the significant peaks at 284.5, 398.5 and 531.2 eV correspond to the binding energies of C_1S_, N_1S_ and O_1S_, respectively. Both the spectra look same as cobalt is not the core element of the NCQDs. The QD simply conjugates with cobalt through non covalent interactions and hence changes its spectral behavior. Energy dispersive X-ray spectroscopy (EDX) was used to analyze the elemental composition of N-CQDs (Fig. S4), which revealed the existence of C, N and O atoms. The structural characteristics of N-CQDs were also confirmed using Raman spectroscopy (Fig. S5). The optical characteristics of N-CQDs were examined using UV–vis and fluorescence spectroscopy. The absorption band at 327 nm in the UV–vis spectra of N-CQDs (Fig. S6) was due to the n-π* transition of the –C–N and C=O/C=N bonds, which may arise from the (–NH_2_) group on the surface of the N-CQDs^20–22^. The aqueous solution of N-CQDs exhibits a brilliant blue color under UV light (365 nm), indicating that the N-CQDs have a blue fluorescence (Fig. S6 inset). It was observed that N-CQDs have a significant emission peak at 430 nm when excited at 350 nm (Fig. S7). From the pH titration experiment, the N-CQDs were shown to be very stable in the pH range of 7–9 (Fig. S8). The quantum yield of N-CQDs (QY) was found to be 27.15%.

#### Mechanism of quenching and enhancement of the N-CQDs/Co complex

The N-CQDs was infused with Co^2+^ solution to make N-CQDs/Co complex. TEM, DLS and FT-IR were used to investigate the formation of the N-CQDs/Co complex. TEM images (Fig. 2A,B) and DLS results (Inset in Fig. 2A,B) clearly illustrated that the N-CQDs were well-dispersed in the absence of Co^2+^, but massive aggregation formed in the presence of Co^2+^. From FT-IR spectra, upon the addition of Co^2+^ ion the sharp ν_OH/NH_ peak shifted from 3433 to 3000 cm^−1^ and became broad (Fig. S2). This implies concrete evidence for the aggregation of N-CQDs by Co^2+^ ensuing the fluorescence quenching. The quenching was caused due to the binding of Co^2+^ on the surface of the N-CQDs resulting them clumped together. The aggregation-based quenching mechanism was also investigated by UV–vis spectroscopy and fluorescence spectroscopy. The fluorescence spectroscopy distinctly executes the quenching behavior of the aggregation. From Fig. S9 we observed that the fluorescence of the N-CQD was almost quenched when Co^2+^ (10^–3^ M) was added to the N-CQD solution. UV–vis spectra (Fig. S10) showed that the characteristic absorption peak of N-CQDs at 327 nm was blue-shifted and a new absorption peak formed at 305 nm to corroborate the aggregation of N-CQDs in presence of Co^2+^. Furthermore, Stern–Volmer^23^ plot was also established to investigate the type of quenching. Depending on the quenching mechanism, the Stern–Volmer plot might be linear or non-linear. There are three types of quenching namely (i) static quenching (ii) dynamic quenching and (iii) combined dynamic & static quenching. We can obtain a linear plot for static and dynamic quenching by plotting F_0_/F vs. [Q], where F_0_ and F are the fluorescence intensities in the absence and presence of the quencher, respectively and Q is the concentration of the quencher, which was observed in our case (Fig. S11), but an upward (positive) curvature should be obtained in cases of combined dynamic & static quenching, which was not the scenario in our case. Therefore, either a static process or a dynamic mechanism might have quenched the fluorescence. Not the ground state, but the excited states of N-CQDs are impacted by the dynamic quenching process. Therefore, it is unlikely that dynamic quenching will cause the absorbance spectra to change. The absorbance spectra of N-CQDs are expected to change when a complex forms in the ground state during static quenching. Co^2+^ interacts with N-CQDs to form a complex with an absorption spectrum that differ from 305 to 327 nm of N-CQDs itself (Fig. S10). Time-resolved spectroscopic study was also supported the static quenching over dynamic quenching. Fig. S12 shows that the average lifetime of the N-CQDs was unchanged by the addition of Co^2+^ indicating a static quenching mechanism. So, the linear Stern–Volmer plot indicates a static quenching mechanism rather than dynamic quenching.

Interestingly we observed that the fluorescence quenching was recovered after the addition of DCA. We theorized that DCA would bind to Co^2+^ and scavenge Co^2+^ from N-CQDs aggregates. As a result, the DCA/Co complex would disaggregate the aggregated N-CQDs, allowing the N-CQDs to regain their fluorescence. The disassembly of N-CQDs triggered by DCA was confirmed by TEM (Fig. 2C) and DLS (Inset of Fig. 2C). The fluorescence emission changes of the disaggregation process were shown in Fig. 2D. The emission intensity of the aggregated N-CQDs was exceedingly low (Fig. 2D (1)), however, it was increased by 70% after the addition of DCA (Fig. 2D (2)). The clear blue emission was seen with the naked eye under the UV light (Fig. 2D, inset). The shifting of absorption spectra from 305 to 314 nm in UV–vis spectroscopy (Fig. S13) also confirmed the decomplexation of DCA/Co. These results demonstrated that the increased fluorescence intensity of N-CQDs was due to the disaggregation-induced enhancement (DIE) by DCA. The aggregation and disaggregation process occur in ground states which is evident in TCSPC results (Fig. S12, Table S2) as it does not show any changes in life-time neither in N-CQDs/Co complex nor in disaggregated N-CQDs.

#### Fluorescence detection of DCA by N-CQDs/Co complex

**Figure 3:**
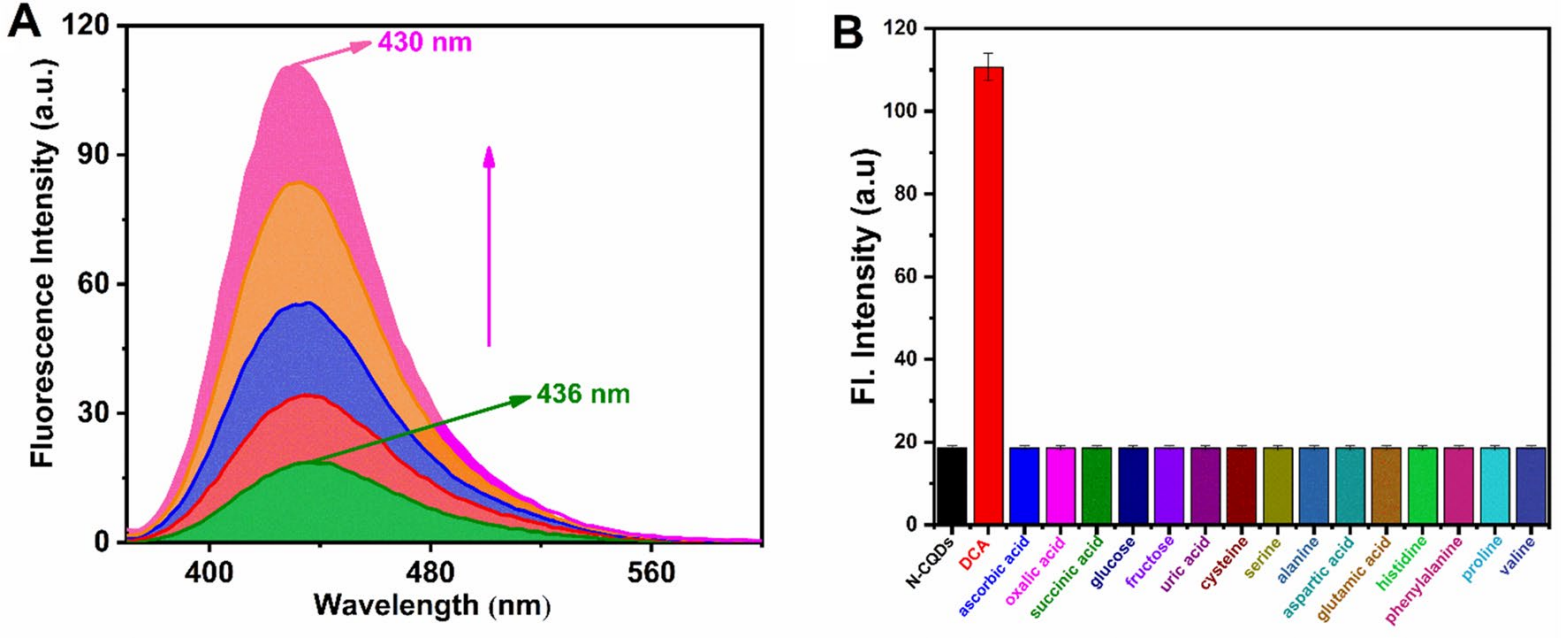
(**A**) Fluorescence emission spectra of the N-CQDs/Co complex in the presence of different concentration of DCA. (**B**) competitive fluorescence spectra of N-CQDs/Co complex with different acids and amino acids at 430 nm (λex= 350 nm). All experiments were performed in PBS buffer (pH=7.4).

To determine the effectiveness of the N-CQDs/Co complex as a sensor, a quantitative detection of DCA based on ‘off–on’ fluorescence mechanism was done by adding various concentrations of DCA to the N-CQDs/Co complex solution and recording the fluorescence response. As demonstrated in Fig. 3A, the fluorescence intensity of the N-CQDs/Co complex increased with increasing DCA concentration and then it reached maximum intensity when DCA concentration was 1000 µl. Regression analysis was used to determine the binding affinity of the N-CQDs/Co complex for DCA, which was determined to be 0.84 × 106 M^−1^ (Fig. S14). A linear relationship between fluorescence intensity and DCA concentration was seen over the range of 2.26–3.34 µM, with a correlation coefficient (R^2^) of 0.9998. The limit of detection (LOD) was found to be 8.7 µM (Fig. S15) based on the formula, 3σ/m (σ is the standard deviation and m is the slope of the curve).

Furthermore, to determine the selectivity of the N-CQDs/Co complex towards DCA, fluorescence changes were recorded in the presence of various acids and amino acids existing in biological system. Except DCA, no fluorescence changes were observed while performing the above experiment indicating that the N-CQDs/Co complex is extremely selective for DCA (Fig. 3B).

### The monitoring of N-CQDs/Co complex on in vitro and in vivo systems

**Figure 4.**
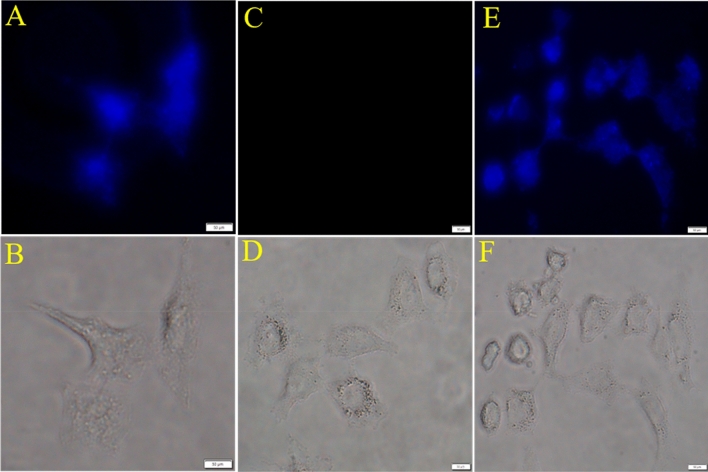
Fluorescence microscopic images of HUH-7 cells treated with N-CQDs, Co^2+^ and DCA (**A**) cell treated with N-CQDs at concentration 10 µg/ml (**B**) phase contrast images of (**A**). (**C**) Cell treated with N-CQDs + Co^2+^ at concentration 10 µg +10 µg/ml (**D**) phase contrast images of (**C**). (**E**) Cell treated with N-CQDs + Co^2+^ + DCA at concentration 10 µg+10 µg+20 µM. (**F**) phase contrast images of (**E**). All images are acquired with 20x objective lens with applied wavelength λex = 340 nm, λem = 459 nm filter used DAPI.

**Figure 5:**
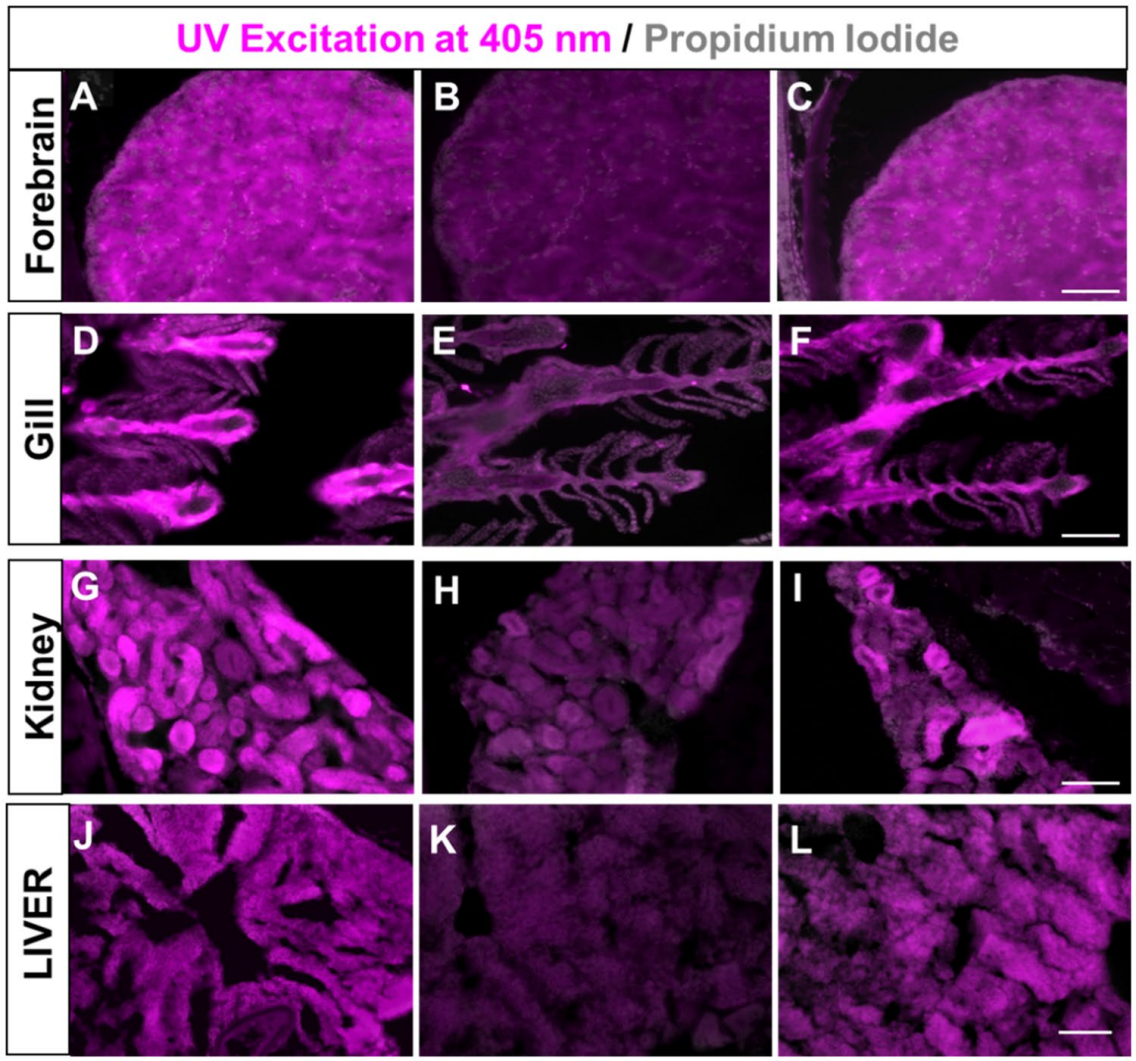
(**A–C**) Fluorescence images of adult zebrafish forebrain after UV excitation at 405 nm showing florescence intensity in neuronal cells after systemic treatment with N-CQDs (**A**), N-CQDs + Co^2+^ (**B**) and N-CQDs + Co^2+^ + DCA (**C**) as mentioned in SI Table 1. (**D-F**) Fluorescence images of adult zebrafish gill after UV excitation at 405 nm showing florescence intensity in primary gill lamellae after systemic treatment as mentioned in (**A–C**). (**G–I**) Fluorescence images of adult zebrafish kidney after UV excitation at 405 nm showing florescence intensity in kidney tubule cells after systemic treatment as mentioned in (**A–C**). (**J–L**) Fluorescence images of zebrafish liver tissue section (30µm) after UV excitation at 405 nm showing florescence intensity after systemic treatment as mention in (**A–C**). Propidium iodide was used to stain nuclei of all tissue sections. Scale bars = 50 µm.

To establish the appropriateness of the N-CQDs/Co complex to detect DCA in biological system, we have detected DCA in human cell line (Fig. 4) and in zebrafish (Fig. 5). Based on the photophysical properties of N-CQDs, it has been found that the human cells and the adult zebrafish neuronal (forebrain) and non-neuronal (gill and kidney) tissues can be labelled with N-CQDs and exhibit strong fluorescence signals under fluorescence microscope. Furthermore, the fluorescence signal of the N-CQDs both in HuH-7 and zebrafish can be quenched upon treatment with Co^2+^ while the signals were again retrieved upon addition of DCA. To achieve this goal, it is necessary to evaluate the cytotoxicity of N-CQDs, Co^2+^, and DCA as well as their complex, such as N-CQDs/Co and N-CQDs/Co + DCA complex, on living cells to their working concentration*.* It was found that there was no detectable cytotoxicity^24, 25^ (Fig S16) in HuH-7 human cell line and adult zebrafish^26–28^.

## Conclusion

In summary, we developed a Cobalt-conjugated carbon quantum dots that can selectively detect DCA via “off–on” mechanism. The intrinsic fluorescence of the N-CQDs can be quenched upon addition of Co^2+^ that form N-CQDs/Co complex and can further be recovered by the addition of DCA due to disaggregation induced enhancement (DIE). The aggregation and disaggregation mechanism were evident by TEM, DLS, UV–vis, fluorescence and FT-IR. The quantum dot was used to check its suitability to detect DCA from the human cell line and live zebrafish. We found that both the N-CQDs/Co complex and DCA can easily enter inside live cells and can be dispersed throughout the body, i.e. forebrain, gills, kidney tissue, etc. of the adult zebrafish once treated with. It has also been found that N-CQDs, N-CQDs/Co complex and DCA are non-cytotoxic to the human cell line and the adult zebrafish. Hence, we propose that this unique N-CQDs can be applied to selectively detect DCA both from environmental sample and from biological live-systems, as well.

### Experimental section

#### Materials and methods

Diethylenetriamine (DETA), Cobalt chloride hexahydrate, Ascorbic acid, salicylic acid, oxalic acids, succinic acid, glucose, uric acid, aspartic acid, cysteine, serine and all other acid group containing amino acids are purchased from Sigma-Aldrich Pvt. Ltd. India. DCA was purchased from TCI India. Here double distilled (dd) water used as solvent. Please note we have moved the section "Ethical declaration" and "Associated consent" to the end of the methods, as per house style.It is absolutely OKAY.

##### Ethics declaration

We confirm that the present study is reported in accordance with ARRIVE guidelines. All animal experiments were carried out according to the CPCSEA, Govt. of India guidelines and protocols are approved by the Institutional Animal Ethics Committee of the University of Calcutta (CPCSEA/ORG/CH/Reg. No 925/298).

##### Associated content

Experiments on adult zebrafish for studying the turn-on in vivo monitoring of DCA shown in Table S1, HRTEM image of N-CQDs, FT-IR spectra of the N-CQDs and N-CQDs/Co complex, Full XPS spectrum of N-CQDs/Co, EDX data of the N-CQDs, Raman spectra of the N-CQDs, UV–vis spectra of the N-CQDs, Fluorescence spectra of the N-CQDs, pH titration study of the N-CQDs, fluorescence titration of N-CQDs with Co^2+^, UV–vis titration of N-CQDs with Co^2+^, UV–vis titration of N-CQDs/Co complex with DCA, Fluorescence lifetime decay curve of N-CQDs and after addition of DCA, binding constant calculation of N-CQDs/Co complex towards DCA, LOD calculation of N-CQDs/Co complex with DCA, Cytotoxicity assay.

### Characterization

The morphology of N-CQDs and Cobalt-conjugated QDs was studied using a transmission electron microscope (HR-TEM JEOL JEM 2100). A Malvern 4800 Autosizer with a 7132 correlator was used to record DLS data. A Nexus TM 870 spectrophotometer was used to acquire Fourier transform Infrared (FT-IR) data. XPS spectral measurement was done by using a PHI 5000 VERSA PROBE III (ULVAC PHI (Physical Electronics), USA), Raman spectra were acquired by using a micro Raman spectrometer (LabRam HR, Horiba Jobin Yvon) with a Peltier-cooled CCD detector, a 785 nm laser was used as an excitation source. A Perkin Elmer Model LS 55 spectrophotometer was used to record the fluorescence spectra. A SHIMADZU UV-3101PC spectrophotometer was used to make UV spectral measurements. Multifunctional time-correlated single-photon counting (TCSPC) fluorescence spectrophotometer (Model 1057, Fluorlog, Horiba Scientific Tech. USA) were used for this experiment. Benchmark—Hermle Z216 MK Refrigerated Benchtop Centrifuge is used to centrifuge. An Olympus IX73 fluorescent microscope was used to capture live cell images. An Olympus BX53 fluorescence microscope was used to capture live zebrafish imaging.

### Synthesis of N-CQDs

A simple microwave assisted approach was used to make nitrogen doped carbon quantum dots (N-CQDs). In a 100 mL conical flask, 0.3 g of ascorbic acid was dissolved in 80 mL double distilled water. After obtaining a clear solution, add 0.6 mL DETA and stir for 3–5 h. After that, the solution mixture was heated for 4 min in a typical microwave oven (560 W). After that, cooled the solution and centrifuge it at 10,000 rpm for 15 min. Finally, use a 0.22 µm membrane filter to filter the solution. The N-CQDs solution was then kept at 4 °C for subsequent application and characterization.

### Synthesis of N-CQDs/Co complex

100 µl of N-CQDs solution were added into 1.9 ml deionized water to make a stock solution. The QD solution was then infused with 950 µl (10^–3^ M) of Co^2+^, which was stirred for 10 min. The fluorescence spectra of the N-CQDs/Co complex were then measured at 350 nm excitation.

### Detection of DCA

In a quartz cuvette, the N-CQDs/Co complex solution was made using a QD stock solution (100 µl of N-CQDs and 1.9 ml dd water) and 950 µl (10^−3^ M) of Co^2+^ solution. After that, varied concentrations of DCA from a 10^−3^ M stock solution were added to the N-CQDs/Co complex solution, and the fluorescence spectra was recorded at 350 nm excitation.

### Quantum yield

The quantum yield (QY) of N-CQDs was calculated using quinine sulphate as reference (QY = 0.54) and The QY of N-CQDs was calculated using the equation below:

1 $${\text{Q}}_{{\text{x}}} = {\text{ Q}}_{{{\text{st}}}} \left( {{\text{I}}_{{\text{x}}} /{\text{I}}_{{{\text{st}}}} } \right) \, \left( {{\text{A}}_{{{\text{st}}}} /{\text{A}}_{{\text{x}}} } \right) \, \left( {{\text{n}}_{{\text{x}}}^{{2}} /{\text{n}}_{{{\text{st}}}}^{{2}} } \right),$$

where ‘Q’ denotes the quantum yield, I is the observed integrated emission intensity, n is refractive index, and A denotes the absorbance at 350 nm. “st” stands for a known QY standard, while “x” stands for the sample. The absorbance value of each solution was kept below 0.05 at the excitation wavelength of 350 nm to minimize reabsorption effects.

### Live cell imaging

#### Cell line and cell culture

##### Cell culture

HuH7 cell lines were created by continuously cultivating cells in Dulbecco’s Modified Eagle’s Medium (DMEM; Sigma Chemical Co., St. Louis, MO) with 10% fetal bovine serum (Invitrogen), 100 μg/mL penicillin, and 100 μg/mL streptomycin added as supplements. Initial cell growth was placed at 37 °C in a CO_2_ incubator with a 5% CO_2_ and 95% air environment in a 75 cm^2^ polystyrene tissue culture flask with a filter lid. Cell density in culture media was adjusted to 1.0 × 10^5^ per/well once the cells entered the logarithmic phase. The cells were then utilized to inoculate in 1.0 mL (1.0 × 10^4^ cells) of cell suspension in each glass bottom dish. The culture medium was taken out after cell adhesion. Phosphate buffered saline (PBS) (pH 7.0) was used to wash the cell layer twice before the appropriate treatment was applied for the experiment.

### Cell imaging study

1 × 10^4^ HuH7 cells in 1000 μL of medium were seeded on three sterile 35 mm glass bottom culture dishes (ibidi GmbH, Germany) and cultured for 10 h at 37 °C in a CO_2_ incubator for fluorescence imaging investigations. After that, cells were washed with 500 mL of DMEM followed by incubation with the N-CQDs (10 μg) dissolved in 1000 μL DMEM at 37 °C for 1 h in a CO_2_ incubator and then washed three times with PBS (pH 7.0) to eliminate any extra N-CQDs while being observed under an Olympus IX73 fluorescent microscope. Images obtained through section scanning were examined using DAPI filter with excitation at 339 nm monochromatic laser beams, and emission spectra were integrated over a range of 459 nm. The cells were once again incubated with Co^2+^ (10 μg) for 20 min and excess Co^2+^ was washed three time with PBS (pH 7.0). The cells were once again treated with DCA (20 μM) for 20 min, and any remaining DCA was removed by PBS for three times (pH 7.0). Using DAPI filters and monochromatic laser beams at 339 nm for excitation, images were taken under a microscope, and emission spectra were integrated over a range of 459 nm. To compare the relative intensity of intracellular fluorescence across all photos, fluorescence microscope settings including transmission density and scan speed were kept constant.

### Cytotoxicity assay

Studies conducted in vitro proved that N-CQDs have good selectivity when it comes to detecting Co^2+^ in biological systems. HuH7 human cancer cell (RRID: CVCL_0336) models were used. To investigate the cytotoxicity of the N-CQDs, Co^2+^ and DCA as well as their complex, such as N-CQDs/Co and N-CQDs/Co + DCA complex, at various concentrations, the well-known MTT assay was used. A cytotoxicity test for each experiment demonstrates that neither the N-CQDs nor the Co^2+^ are cytotoxic to the human cancer cell HuH7, nor do they have a substantial impact on cell viability at the tested concentrations.

Behavioural analysis of all fishes was done to check for any signs of stress, asphyxiation or respiratory distress as depicted by slow swimming, isolation from the group, no interest in feeding and swimming to the surface for air. For chlorine toxicity assessment fishes were checked for signs of gill necrosis and cherry red swollen gills in accordance to the guidelines provided in Zebrafish Information Network (https://zebrafish.org/wiki/health/disease_manual/water_quality_problems). For Cobalt and N-CQDs dosage it was ensured that the working concentrations were lower than the toxicity levels as reported previously.

For assessment of cytotoxicity in zebrafish tissues, TUNEL assay (Terminal deoxynucleotidyl transferase mediated dUTP-biotin nick end labeling) was performed using In Situ Cell Death Detection Kit, Fluorescein (11684795910 Roche) on the forebrain and gills of control (no treatments) and treated zebrafish. As shown in Supplementary Fig. S17,S18 no differences were found in control vs treated tissues. The short duration exposure (1–3 days) of these compounds in the aforementioned concentrations do not appear to be toxic to zebrafish tissue.

### Zebrafish maintenance

Zebrafish were maintained at 28 °C on a 14-h light/10-h dark cycle and the study was carried out in accordance with the recommendation in the guidelines provided by CPCSEA Govt. of India guidelines and protocols are approved by the Institutional Animal Ethics Committee of the University of Calcutta (CPCSEA/ORG/CH/Reg. No 925/298) (Committee for the Purpose of Control and Supervision of Experiments on Animals, Ministry of Environments and Forests, Government of India).

### Chemical treatment

Three experimental conditions were set up using 3–4 months old adult zebrafish for studying the turn-on in vivo monitoring of DCA as shown in Table S1.

1. In experimental condition #I, fishes were placed in glass beakers containing 20 ug/ml quantum dots (N-CQDs) in water and left overnight.
2. In experimental condition #II, fishes were treated with N-CQDs on day 1 and 20 ug/ml N-CQDs quencher Co^2+^ on day 2.
3. In experimental condition #III, fishes were treated with N-CQDs on day 1 and Co^2+^ on day 2. On day 3 fishes were treated in water containing 40 mM DCA for achieving fluorescent recovery.

#### Tissue collection & sectioning and microscopy

Fishes were anaesthetized for 5 min in 0.02% tricaine (MS222; Sigma, St. Louis, MO). Zebrafish brain, gill, and kidney were collected and processed according to the protocol of Hui et al.^28^. Briefly the organs were fixed in 4% paraformaldehyde solution O/N at 4 °C and cryoprotected in 20% sucrose incubating overnight. The tissues were embedded in tissue freezing medium and 30 µm thin sections were cut using a cryostat (Leica, 3050S).

For microscopy sections were briefly washed in Phosphate Buffered Saline + Tween 20 (PBT) and counterstained using Propidium iodide (1:100). The slides were mounted and imaged under the Olympus BX53 fluorescence microscope at 405 nm. The blue (N-CQDs) and red (Propidium Iodide) fluorescence as shown in the fluorescence microscope were represented with magenta and grey color respectively in Fig. 5 for the better visualization of color combinations by the color vision deficiency people.

## Supplementary Information

Supplementary Information.

## Data Availability

All data supporting this study and its findings are available within the article and its Supplementary Information or from the corresponding authors upon request**.**
