# Supplementary material for: Cobalt-conjugated carbon quantum dots for in vivo monitoring of the pyruvate dehydrogenase kinase inhibitor drug dichloroacetic acid
Source: Sci Rep. 2022 Nov 12;12:19366. doi: 10.1038/s41598-022-22039-w (PMC9653503; doi:10.1038/s41598-022-22039-w)
Supplement: Supplementary file 1 — Supplementary Information. [file 41598_2022_22039_MOESM1_ESM.doc]

**SUPPORTING INFORMATION**

**Cobalt-conjugated carbon quantum dots for *in vivo* monitoring of the pyruvate dehydrogenase kinase inhibitor drug dichloroacetic acid**

*Jiko Raut^a^, Md Majharul Islam^b^, Rinchen D. Sherpa^c^, Biraj Sarkar^b^, Shanti M. Mandal^d^, Subhra P. Hui^c^, Sukhendu Mandal^b^ and Prithidipa Sahoo*^a^*

^a^Department of Chemistry, Visva-Bharati University, Santiniketan-731235, India.
 Email: [prithidipa@hotmail.com](mailto:prithidipa@hotmail.com)

^b^Department of Microbiology, University of Calcutta, Kolkata-700019 India

^c^S. N. Pradhan Centre for Neurosciences, University of Calcutta, Kolkata-700019, India

^d^Central Research Facility, Indian Institute of Technology Kharagpur, 721302, India

*Correspondence to: Prithidipa Sahoo (Email: [prithidipa@hotmail.com](mailto:prithidipa@hotmail.com))

Number of Page: 14

Number of Tables: 2

Number of Figures: 18

**Table S1**

| **Experimental condition** | **Day 1** | **Day 2** | **Day 3** | **Day 4** |
| --- | --- | --- | --- | --- |
| **I** | **N-CQDs** | **Tissue collection** |  |  |
| **II** | **N-CQDs** | **Co^2+^** | **Tissue collection** |  |
| **III** | **N-CQDs** | **Co^2+^** | **DCA** | **Tissue collection** |

1. **HRTEM image of the N-CQDs**

**
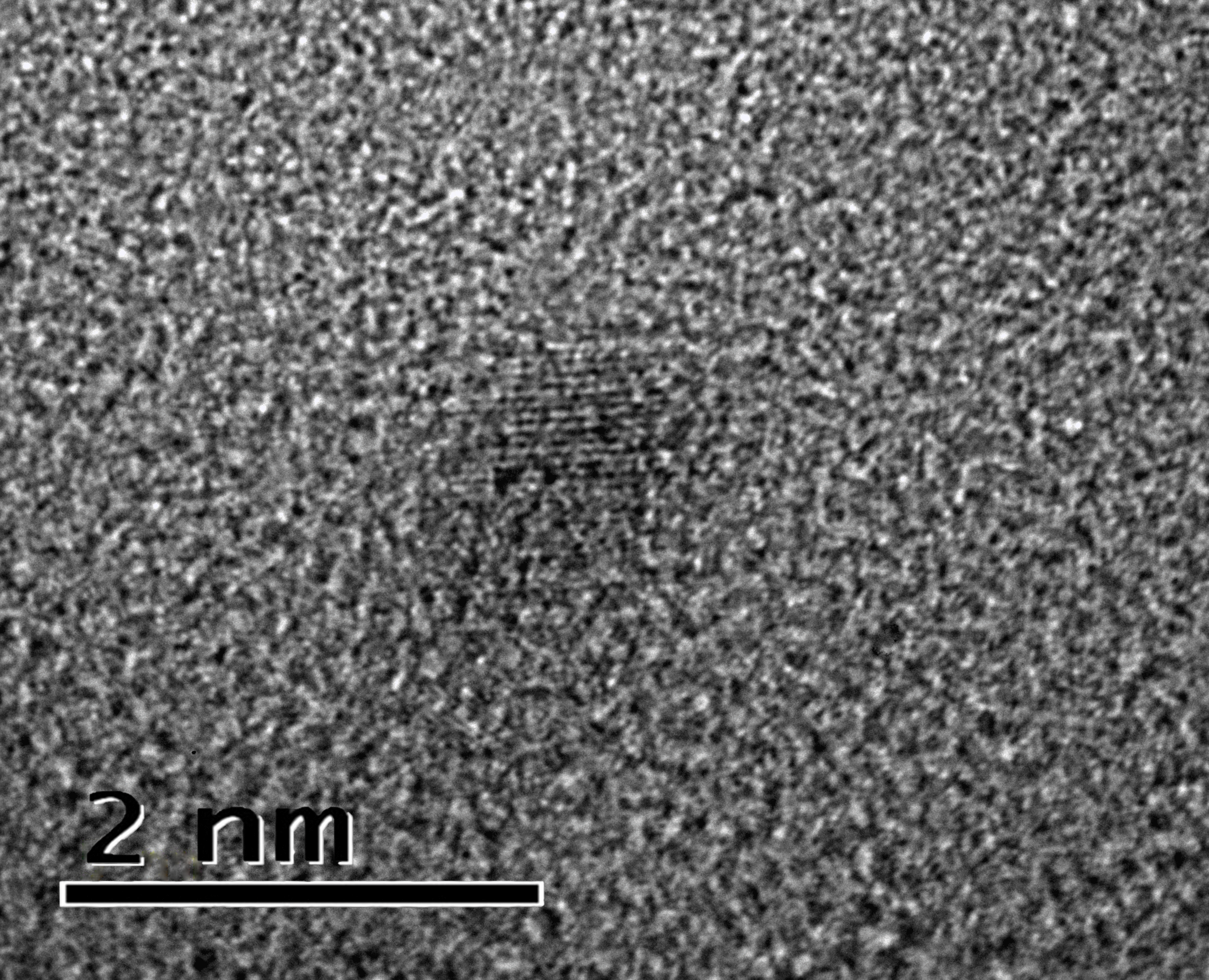
**

**Figure S1.** HRTEM image of the N-CQDs

1. **FT-IR:**
   The peaks at 3433 cm^-1^ and 2932 cm^-1^ are caused by the stretching vibrations of -OH/-NH and C-H, respectively. At 2065 cm^-1^, a C=N stretching vibration occurs. The peak at 1634 cm^-1^ represents the stretching vibration of the C=O bond in the amide group. The C-N peak at 1395 cm^-1^ correlates to nitrogen doping on N-CQDs, implying nitrogen doping. At 1264 cm^-1^, 805 cm^-1^, and 978 cm^-1^, 635 cm^-1^, respectively, C-O stretching vibrations, N-H bending vibrations, and C-H bending vibrations occur.


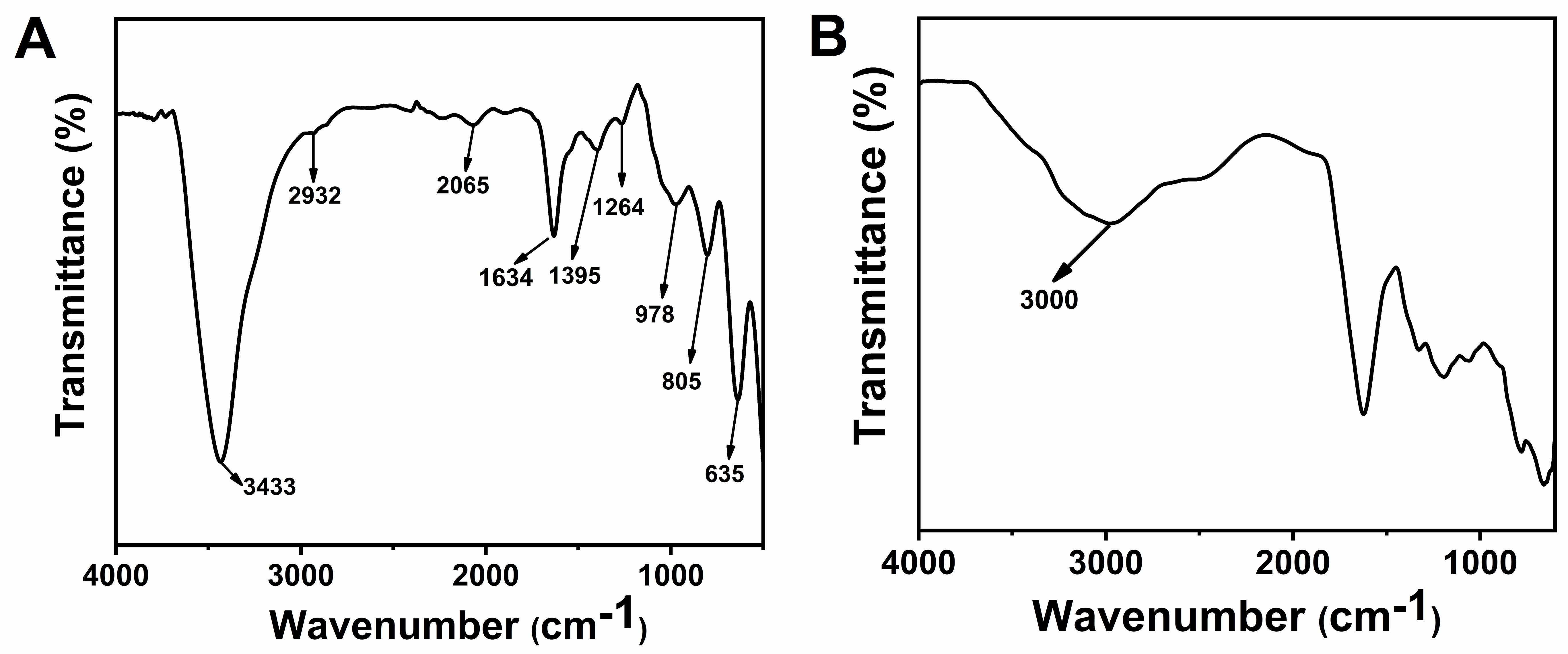


**Figure S2.** (A) FT-IR spectrum of the synthesized N-CQDs and (B) FT-IR spectrum of the N-CQDs/Co complex.

1. **XPS Spectrum:**

**
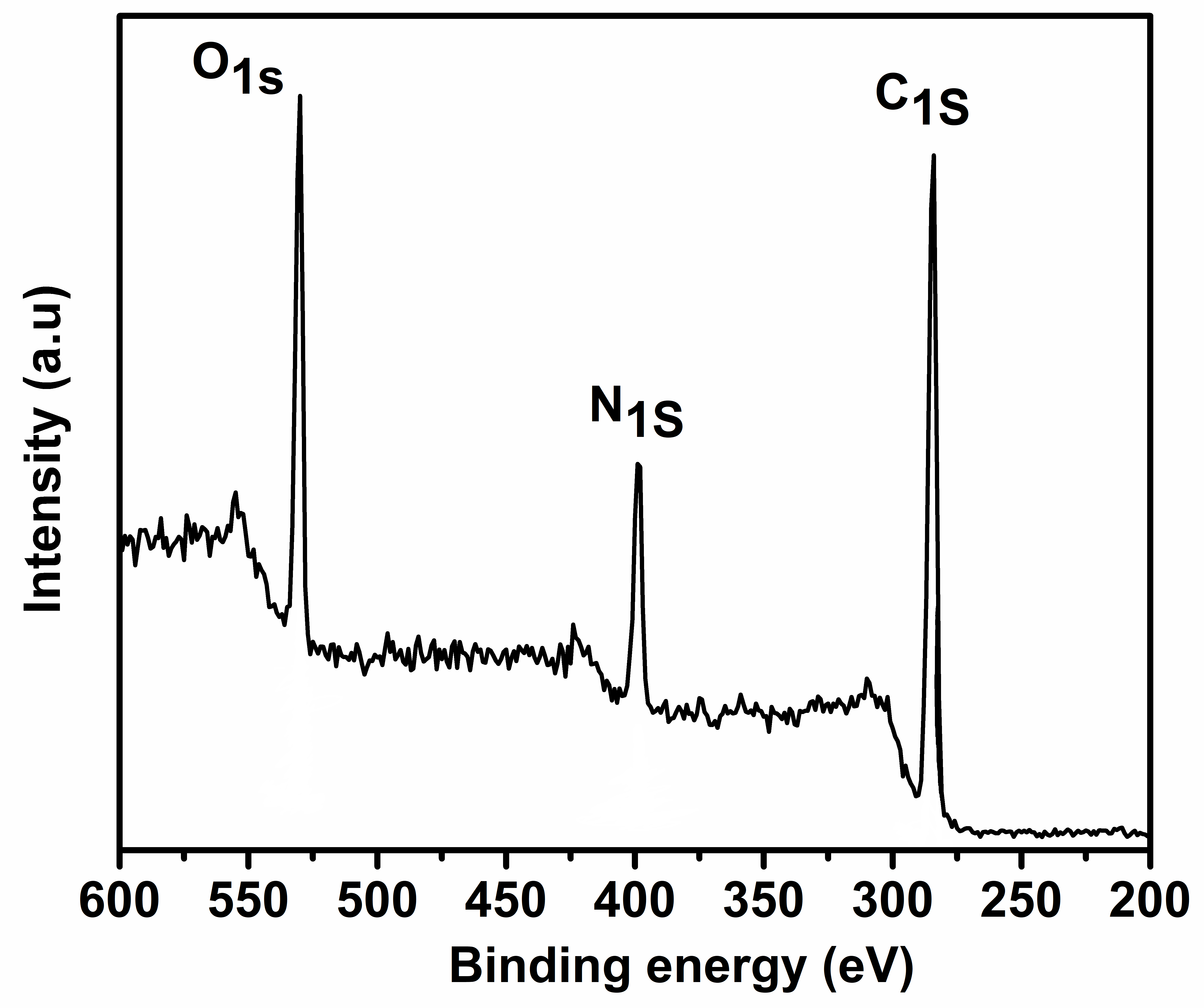
**

**Figure S3.** Full XPS spectra of N-CQDs/Co complex.

1. **EDX DATA**


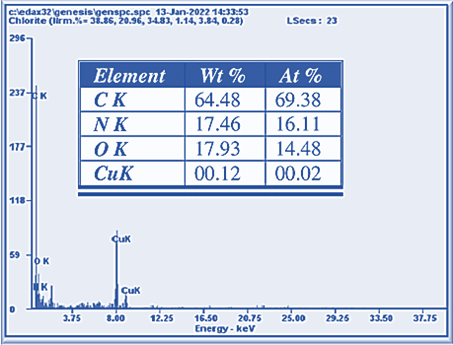


**Figure S4.** EDX data of N-CQDs

1. **Raman spectra**

**
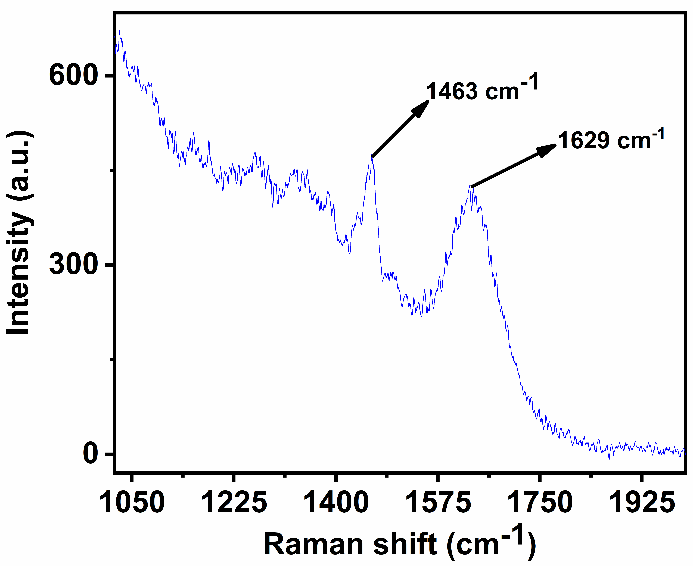
**

**Figure S5.** A detailed examination of the Raman spectra obtained from the synthesised N-CQDs. The D-band and G-band peaks are seen at 1463 cm-1 and 1629 cm-1, respectively.

1. **UV-vis spectrum of N-CQDs**


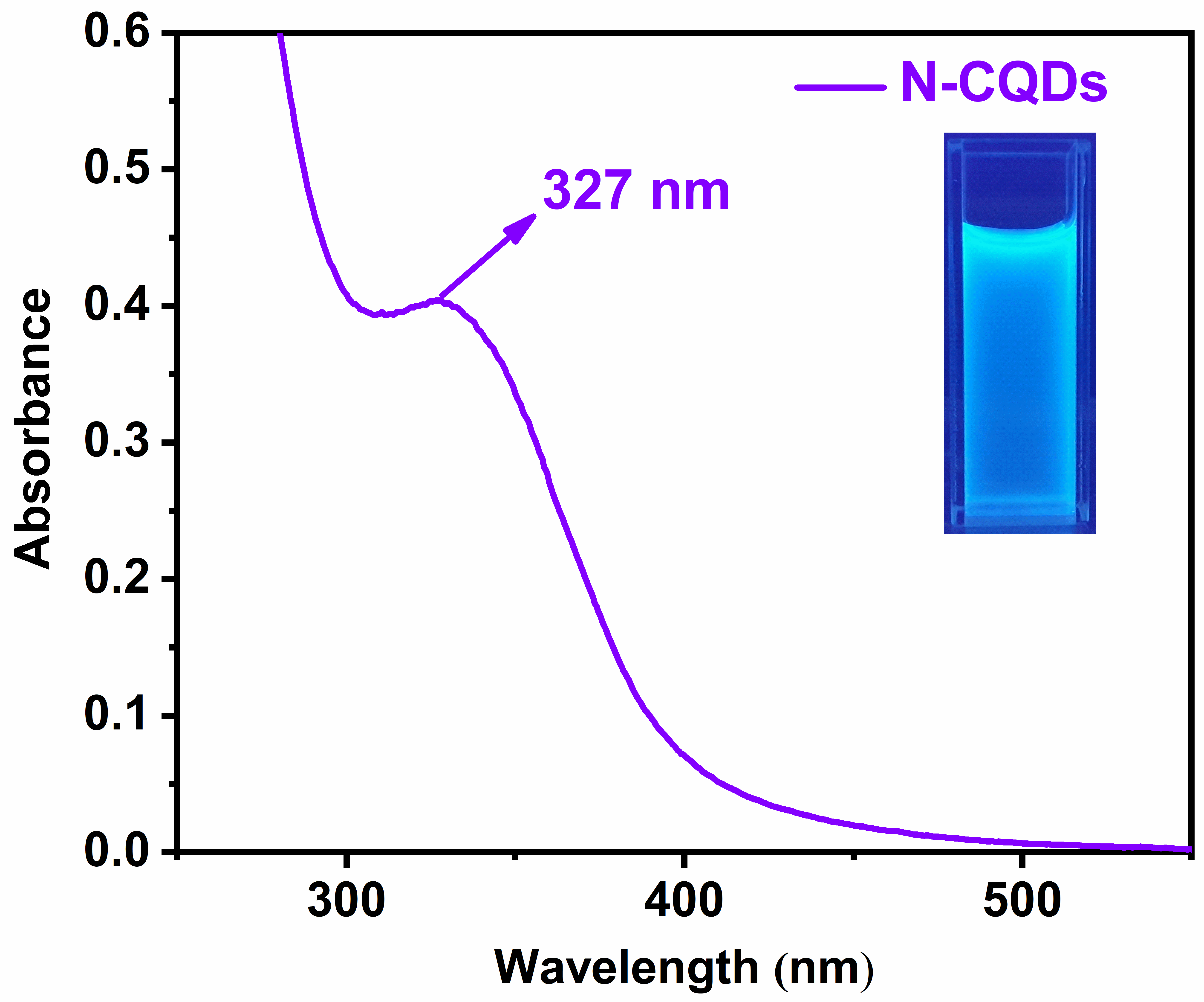


**Figure S6.** UV-vis spectrum of the synthesized N-CQDs (Inset: bright blue colour

of the N-CQDs under UV lamp (365 nm)

1. **Fluorescence spectrum of N-CQDs**


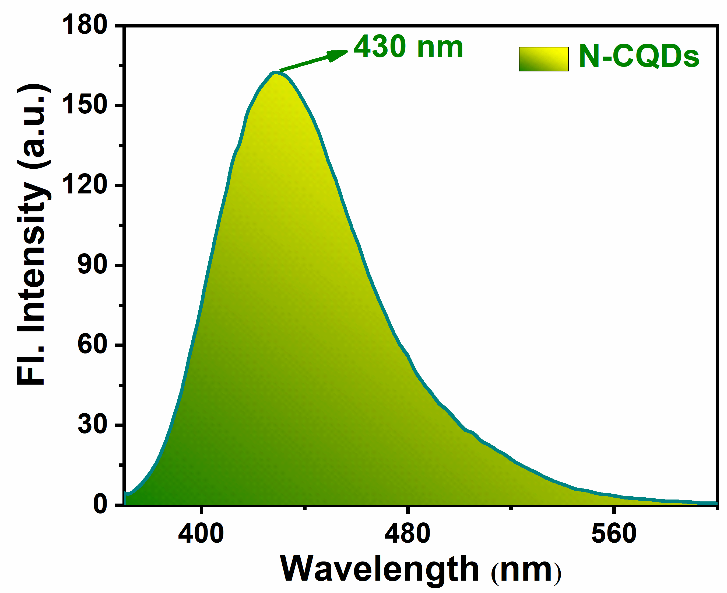


**Figure S7.** Fluorescence spectrum of the prepared N-CQDs with an emission peak

at 430 nm.

1. **P^H^ titration study**


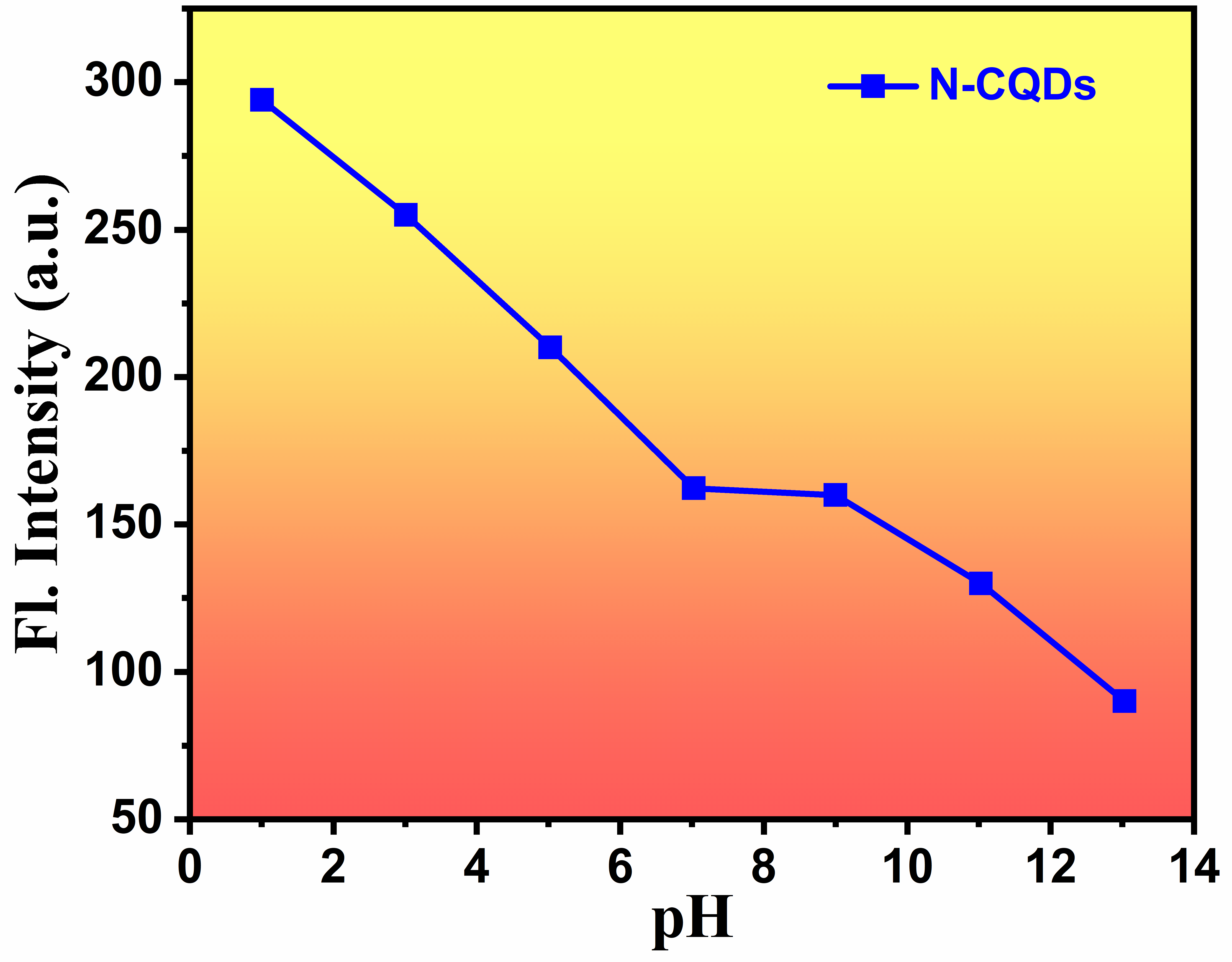


**Figure S8.** p^H^ response study of the N-CQDs

1. **Fluorescence Titration of N-CQDs with Co^2+^**


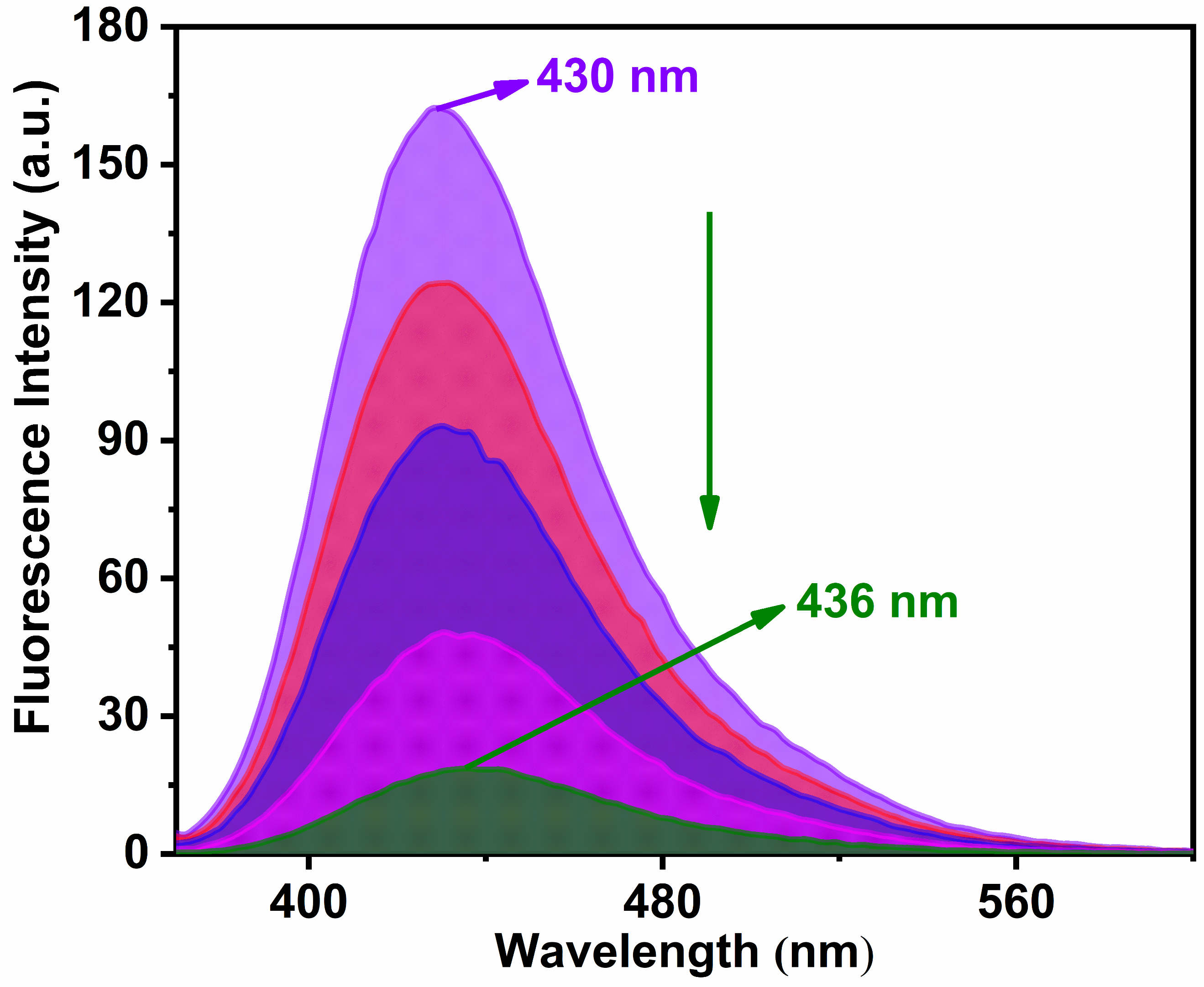


**Figure S9.** Fluorescence of N-CQDs quenched by the addition of different concentration of Co^2+^ (10^-3^ M) at 350 nm excitation.

1. **UV-vis titration of N-CQDs with Co^2+^**


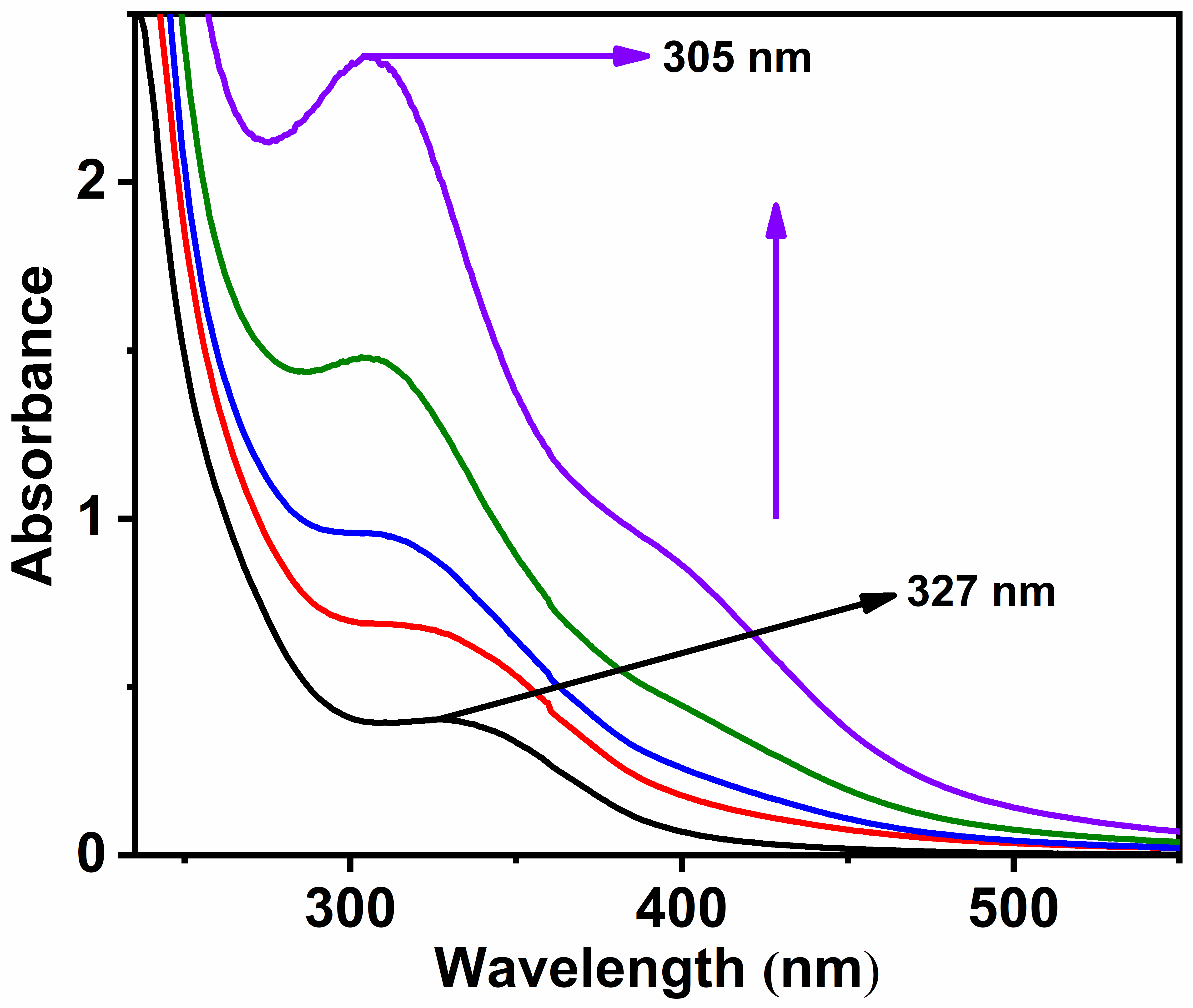


**Figure S10.** UV-vis absorption spectra of N-CQDs upon addition of Co^2+^ (10^-3^ M).
All the experiments were done in PBS buffer (pH=7.4).

1. **Stern-Volmer plot**


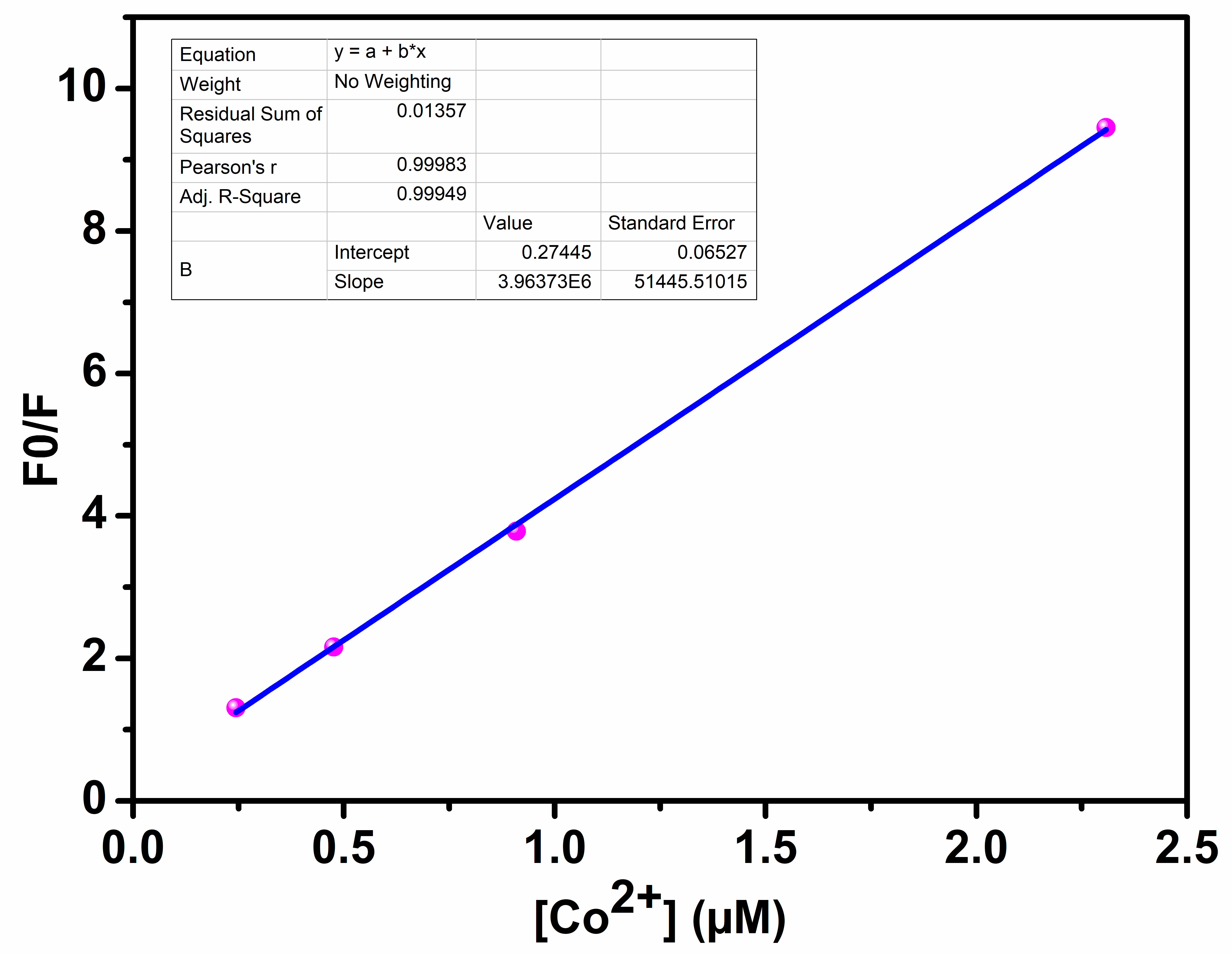


**Figure S11.** Stern-Volmer plot displays the quenching efficiency of Co^2+^

1. **Fluorescence lifetime decay**


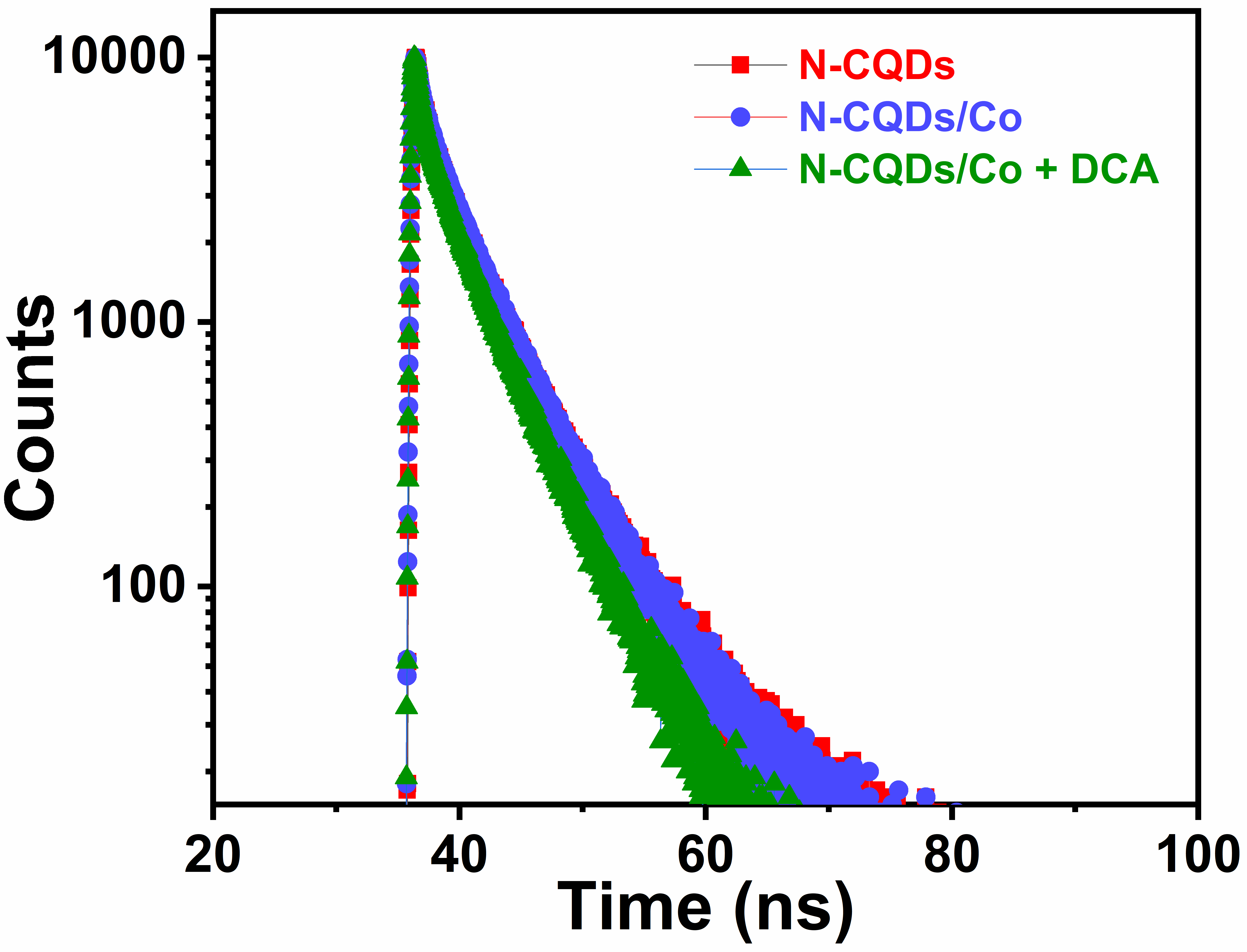


**Figure S12.** The fluorescence lifetime of the N-CQDs, N-CQDs/Co and N-CQDs/Co + DCA.

**Table S2. Decay time components of N-CQDs, N-CQDs/Co and N-CQDs/Co + DCA**

| **System** | **b_1_** | **τ_1_** | **b_2_** | **τ_2_** | **b_3_** | **τ_3_** | **<τ>=b_1_τ_1_+b_2_τ_2_+b_3_τ_3_** |
| --- | --- | --- | --- | --- | --- | --- | --- |
| N-CQDs | 4.299193E-02 | 1.985002E-10 | 0.4950652 | 3.943118E-11 | 0.0187228 | 2.160863E-09 | 0.06 ns |
| N-CQDs/Co | 0.2286366 | 6.174627E-11 | 5.986068E-02 | 1.682314E-10 | 2.075233E-02 | 2.117853E-09 | 0.06 ns |
| N-CQDs/Co + DCA | -0.105638 | 1.420313E-09 | 0.3674215 | 7.223522E-11 | 0.1202749 | 1.512267E-09 | 0.058 ns |

1. **UV-vis titration of N-CQDs/Co complex with DCA**


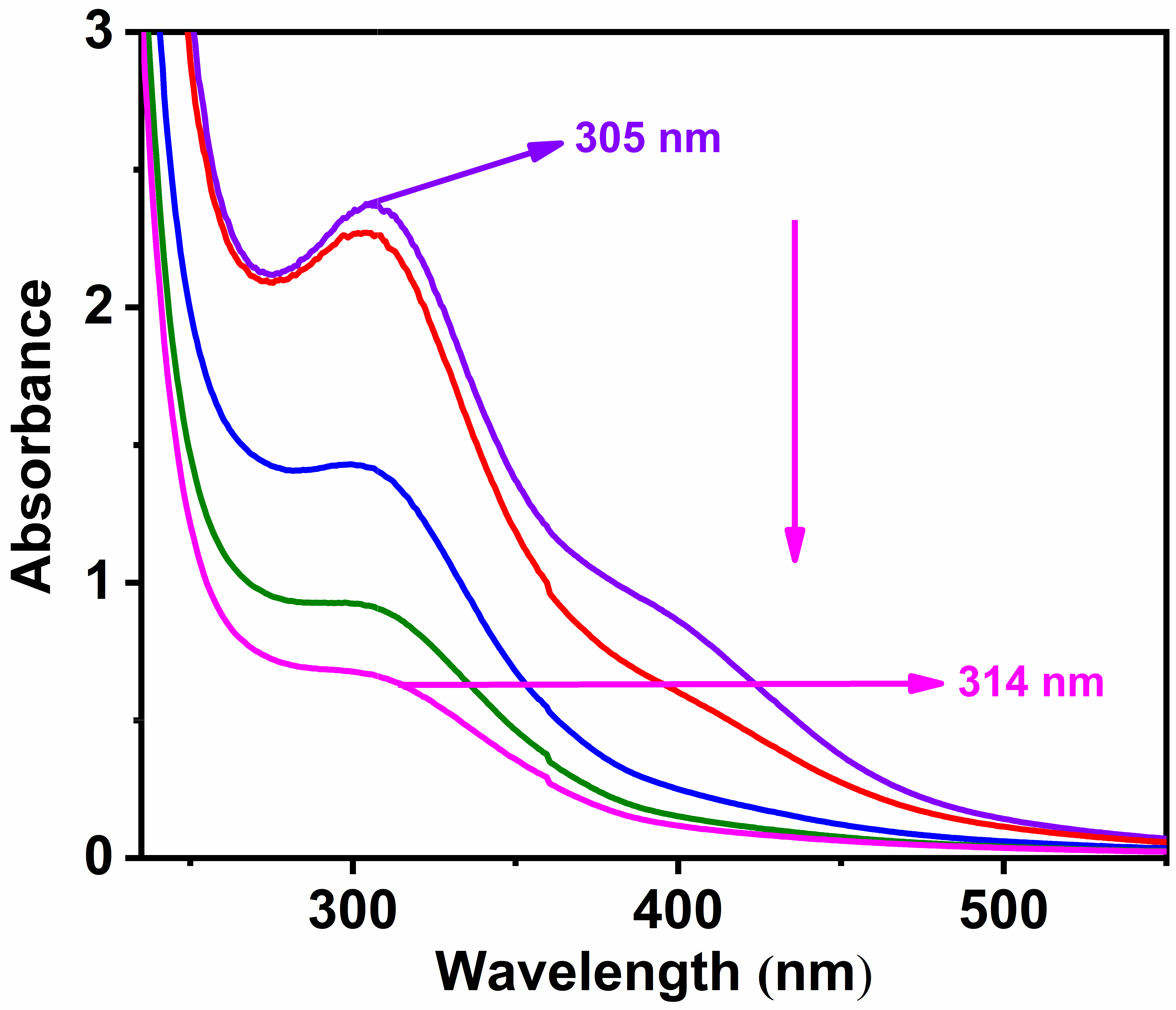


**Figure S13.** UV-vis absorption spectra of N-CQDs/Co complex upon addition

of DCA (10^-3^ M). All the experiments were done in PBS buffer (pH=7.4).

1. **Binding constant calculation graph (Fluorescence method):**


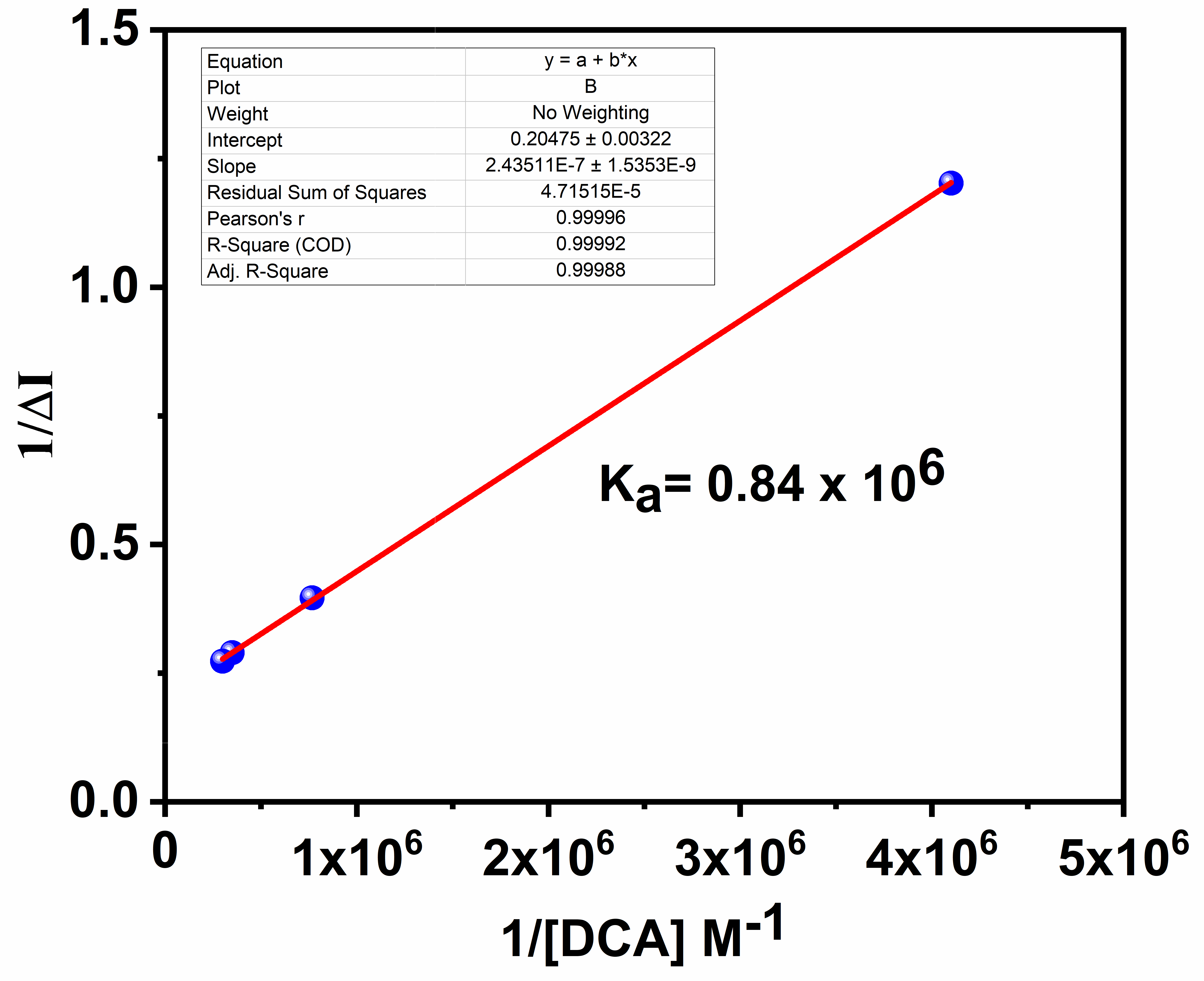


**Figure S14.** Linear regression analysis for the calculation of association constant value by fluorescence titration method. The association const. (K_a_) of N-CQDs/Co complex for sensing DCA was determined from the equation:
K_a_ = intercept/slope. From the linear fit graph, we get intercept=0.20475, slope
 =2.43511 **×**10^-7^. Thus, we get **K_a_**= (0.20475) / (2.43511 **×**10^-7^) = **0.84 × 10^6^ M^-1^**

1. **Calculation of limit of detection (LOD) of N-CQDs/Co complex with DCA:**


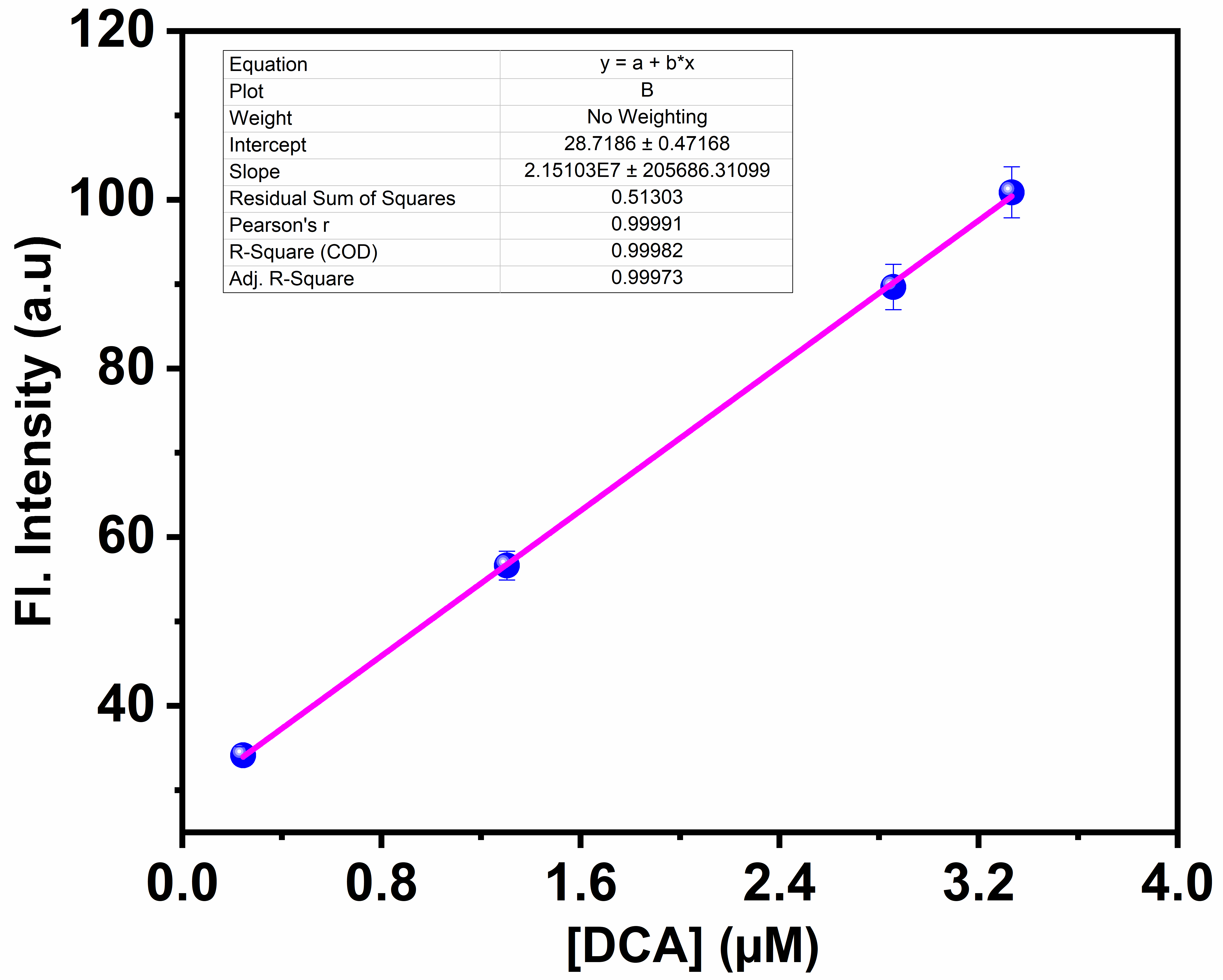


**Figure S15.** Linear fit curve of **N-CQDs/Co** complex at 430 nm with respect to DCA concentration. Standard deviations are represented by error bar (n=3).

From the linear fit graph, we get slope = 2.15103 × 10^7^, and SD (σ) value is 0.62527

LOD for DCA = 3σ/m = (3 × 0.62527)/ (2.15103 × 10^7^) = 8.7 × 10^-6^ M = 8.7 µM

1. **Cytotoxicity Assay:**


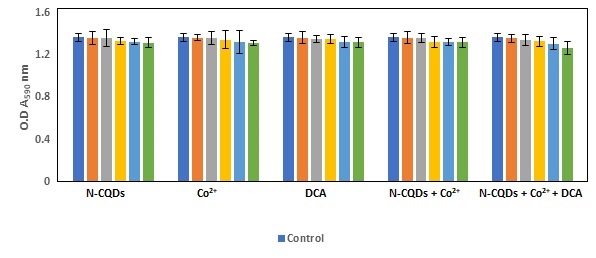


**Figure S16.** MTT assay to determine the cytotoxic effect of complex on **N-CQDs** (0,5,10,15,20,30 μg/ml), **Co^2+^** (0,5,10,15,20,30 μg/ml), and **DCA** (0,5,10,20,30,40 μM), (respective amounts of and **N-CQDs**, **Co^2+^** and **DCA** added to the mixture) HuH7 cells (Human cancer cell HuH7).

1. **Analysis of cytotoxicity in zebrafish forebrain tissue:**


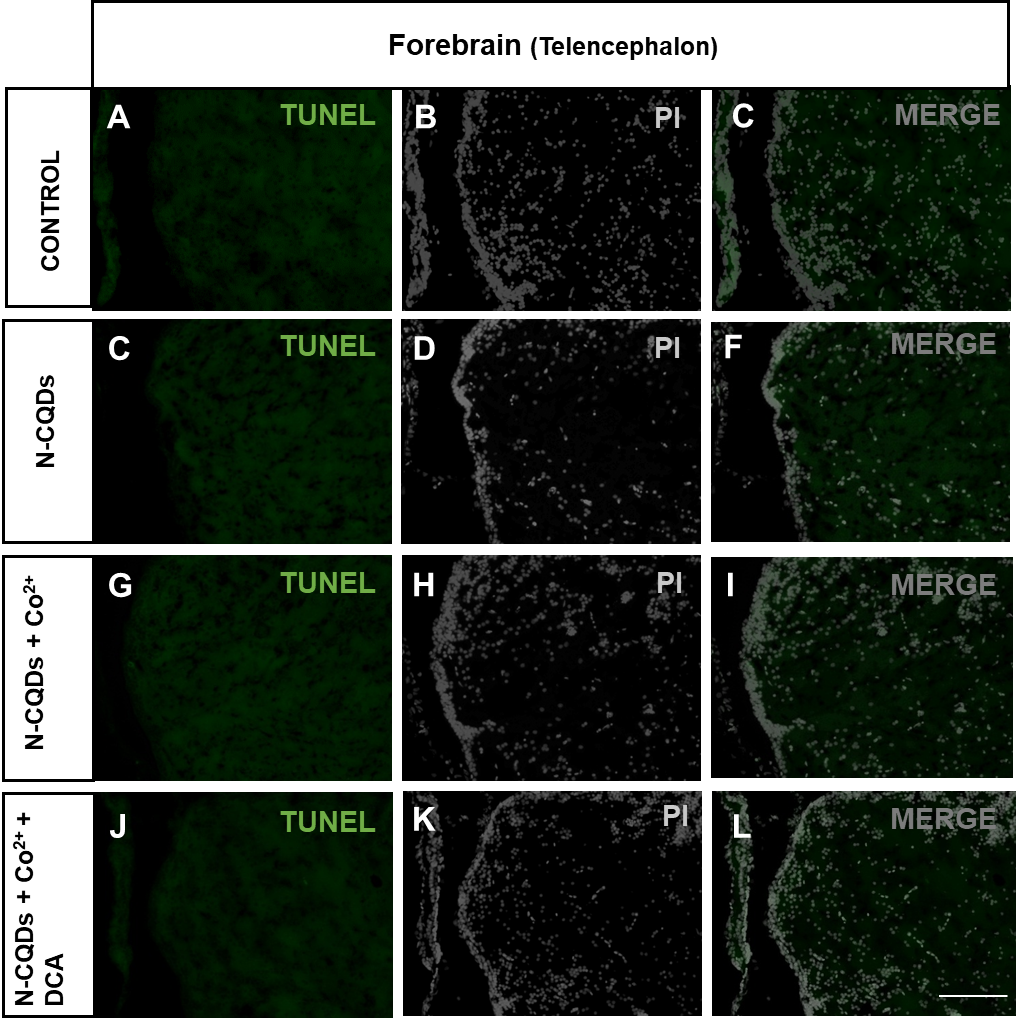


**Figure S17.** Analysis of cell death by TUNEL assay in zebrafish forebrain (telencephalon) to analyse cytotoxicity; A-C: No treatment, D-F: N-CQDs, G-I: N-CQDs + Co^2+^ J-L: N-CQDs + Co^2+^ + DCA.

1. **Analysis of cytotoxicity in zebrafish gill tissue:**


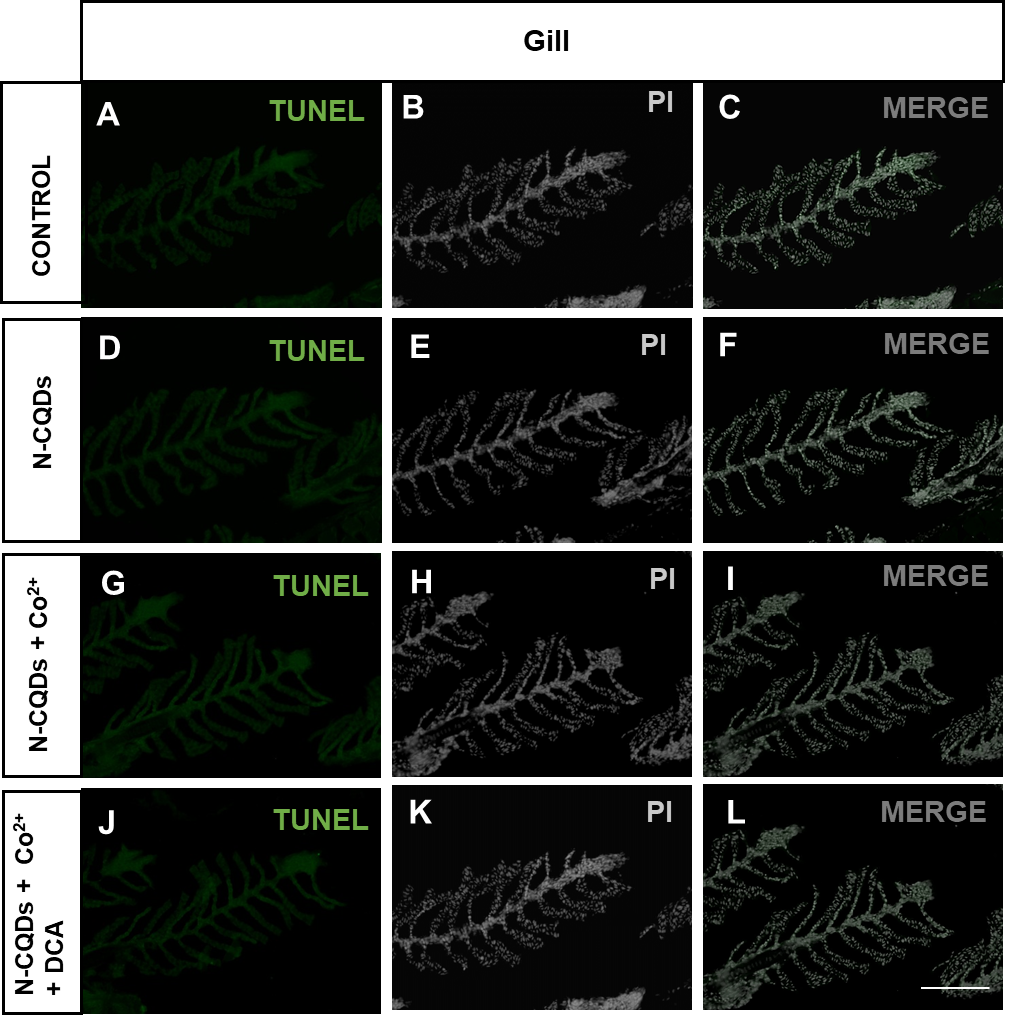


**Figure S18.** Analysis of cell death by TUNEL assay in zebrafish gill to analyse cytotoxicity; **A-C:** No treatment, **D-F:** N-CQDs, **G-I:** N-CQDs + Co^2+^ **J-L:** N-CQDs + Co^2+^ + DCA.
